# Bovine leukemia virus linked to breast cancer in Australian women and identified before breast cancer development

**DOI:** 10.1371/journal.pone.0179367

**Published:** 2017-06-22

**Authors:** Gertrude C. Buehring, HuaMin Shen, Daniel A. Schwartz, James S. Lawson

**Affiliations:** 1 School of Public Health, University of California, Berkeley, California, United States of America; 2 Department of Biotechnology and Biomolecular Science, University of New South Wales, Sydney, New South Wales, Australia; University of North Carolina at Chapel Hill School of Medicine, UNITED STATES

## Abstract

Bovine leukemia virus (BLV), a common virus of cattle globally, was believed for decades not to infect humans. More recent techniques (in situ PCR and DNA sequencing) enabled detection of BLV in human breast tissue, and determination of its significant association with breast cancer in a US population. Using similar techniques to study 96 Australian women, we report here detection of retrotranscribed BLV DNA in breast tissue of 40/50(80%) of women with breast cancer versus 19/46(41%) of women with no history of breast cancer, indicating an age-adjusted odds ratio and confidence interval of 4.72(1.71–13.05). These results corroborate the findings of the previous study of US women with an even higher odds ratio for the Australian population. For 48 of the subjects, paired breast tissue samples, removed 3–10 years apart in two unrelated procedures, were available. For 23/31 (74%) of these, in which the first specimen was diagnosed as nonmalignant (benign or premalignant) and the second as malignant, BLV was already present in benign breast tissue years 3–10 years before the malignancy was diagnosed. This is consistent with the supposition of a causative temporal relationship between BLV infection and subsequent development of cancer.

## Introduction

Australia has a high female breast cancer incidence. Together with New Zealand, it ranks third in regional age-standardized per capita incidence globally [1]. Incidence of breast cancer in New South Wales, where one third of the Australian population resides, has been rising steadily since 1983 [1]. Although case discovery by mammographic screening accounts for some of the increase, a large proportion of this rise is attributed to environmental factors [2]. Studies of descendants of Asian immigrants to countries with a Western culture, revealed rates of breast cancer increasing with each generation [3], leading to the hypothesis that changes in reproductive patterns, use of exogenous female hormones, and/or diet contributed to increased breast cancer incidence. Global statistics indicate a striking geographical correlation between breast cancer incidence and milk consumption [4,5]. Recent studies demonstrated that the DNA of retrotranscribed bovine leukemia virus (BLV) was present in breast tissue of women in the United States [6] and associated with breast cancer [7]. Our goals in this study were to: 1) investigate whether the same is true for women in Australia, a nation with high breast cancer incidence and a diet rich in beef and dairy products; 2) to establish whether BLV presence in breast tissue precedes the development of breast cancer by examining paired tissue specimens from individual subjects surgically removed 3–10 years apart, with the first specimen having no evidence of malignancy.

## Materials and methods

Archived formalin fixed paraffin embedded (FFPE) breast tissue specimens, surgically removed 1995–2010, were acquired from the archives of Douglass Hanly Moir-Pathology, a large pathology service in Macquerie Park, New South Wales, Australia. Humans subjects protocol was approved by the Human Research Ethics Committee of the University of New South Wales, Sydney, Australia and the Committee for the Protection of Human Subjects of the University of California, Berkeley. Pathology reports provided information for all subjects on date and type of surgery, age of patient at time of surgery, and the microscopic diagnosis of the specimen. No information was available on race/ethnicity of subjects. Subjects were selected chronologically, starting with the most recent patients undergoing breast cancer surgery and checking the computer base for previous breast surgeries on the same patient which resulted in a normal/benign diagnosis. Of 102 subjects selected, 6 were eliminated due to small tissue size and/or lack of mammary epithelial cells in the specimen. Among the remaining 96 subjects whose surgeries occurred 1996–2013, 64 had paired specimens with the first removed 3–10 years before the most recent surgery. Samples from 16 of these subjects could not be used in the paired specimen analysis because the earlier specimen was too small for analysis or the interval between surgeries was <3 years. However, the later specimen from these excluded pairs were included in the overall case-control analysis of the association of BLV with breast cancer. Tissue sections (5μ thick) were cut by Douglass Hanly Moir Pathology and shipped to the University of California, Berkeley for the PCR-in situ hybridization assay. The three pathology categories used in study analysis were based on microscopic diagnosis: malignant, pre-malignant (carcinoma in situ, atypical hyperplasia), and benign (changes with low association with subsequent malignancies, e.g. fibrocystic changes, flat epithelial atypia) [8].

Polymerase chain reaction in situ hybridization (PCR-ISH), adapted from Nuovo [9], was used to identify which cell types within tissues were BLV positive. FFPE sections were received on super adherent glass microscope slides and deparaffinized through standard series of xylene, ethanol, and water. To enable entry of PCR mix into cells, specimens were permeabilized with 2mg/mL pepsin in 0.1 N HCl, 50–55 min and smears of positive and negative control cells, 20 min, then pepsin inactivated with (100 mmol/L Tris-HCl, 100mmol/L NaCl, pH 7.4) applied for 1 min, followed by a Dulbecco’s phosphate buffered saline (DPBS) rinse and 5 min in absolute ethanol. Samples were surrounded with a 15 x 15mm frame seal chamber (Bio-Rad Hercules, CA, USA), 60μL of PCR mix placed into the chamber, and the plastic cover sealed over the frame. The PCR mixture was 4.0mmol/L MgCl_2_, 0.4 mmol/L dNTPs, 1 μmol/L primers (Operon Biotechnologies, Huntsville, AL, USA), 0.06% bovine serum albumin (BSA) (free of BLV and antibodies to BLV), and 0.053 U/μL Amplitaq Gold DNA Polymerase (Applied Biosystems, Foster City, CA, USA). The positive control was the FLK (fetal lamb kidney) cell line, stably infected with and replicating BLV [10], and the negative control was Tb_1_Lu, a bat lung cell line [11], known to be uninfected with BLV. The species of origin of both cell lines were authenticated using species specific cytochrome oxidase primers [12]. DPBS suspensions (≈50μL) of control cell lines were deposited on super adherent glass slides, dried, and fixed 18 hours in buffered neutral formalin. Primer sequences, below, were from the *tax* region of the BLV genome. Their genomic location is shown in base pair (bp) numbering according to GenBank accession #EF600696.

Forward (bp 7310–7329): ATGTCACCATCGATGCCTGG

Reverse (bp 7423–7404): CATCGGCGGTCCAGTTGATA

BLV specificity of these primers was evidenced by strong sequence alignments [13] with BLV (E = 0.23), and lack of significant alignment with any sequences of the human genome, including human endogenous retroviruses (E = 16.0 for forward and 255.0 for reverse primer). The E value is a measure of chance similarity with values less than zero having high homology and values greater than zero having low homology with the sequence being queried [13]. Further confirmation of primer specificity was obtained by previous laboratory experiments that demonstrated these same primers amplified BLV but failed to amplify other viruses that were representatives of all oncogenic retroviral families, lentiviruses, as well as viruses reported present in human breast tissues (human endogenous retrovirus-K, human papillomaviruses 16, 18, Epstein-Barr virus, cytomegalovirus, and human herpes viruses 1, 2) [6]. Slides were placed into an in situ PCR machine (Hybaid Thermo OmniSlide; Cambridge Biosystems, Cambridge, UK) for amplification. Cycling parameters were: 1 cycle of 93°C for 10 min, 57°C for 1.5 min, then 30 cycles of 92°C for 30 seconds, 57°C for 1.5 min, and 69°C for 2 min, followed by final extension at 69°C for 10 min.

Probe labeling for ISH was performed with the PCR Dig Probe Synthesis Kit (Roche) according to manufacturer’s instructions, including recommended adjustments for short probes (<1kb) with high GC content. Template DNA was from FLK cells. The reaction mixture was: 5 μL 10X concentration of the provided buffer containing MgCl_2_ and an additional 1μl of 25mM MgCl_2_ (to optimize for the specific primers used in these experiments), 2.5 μL DIG probe synthesis mix, 2.5 μL stock dNTPs, 0.5 μM of each primer, 0.75 μL kit enzyme mix, 200 ng template DNA, and PCR grade water added up to total volume of 50μL. Cycling parameters were: 1 cycle of 95°C for 2 min, then 33 cycles of 95°C for 30 sec, 56°C for 30 sec, and 72°C for 45 seconds, followed by final extension at 72°C for 7min. PCR reaction was performed in a conventional thermocycler. Before use, the amplified fragments were visualized in 2% agarose with 0.5μg/ml ethidium bromide.

PCR-ISH mixture was prepared as a 1:4 ratio of probe to the probe buffer. Slides were rinsed once in PBS, placed in 2X SSC (1.753 g NaCl, 0.82 g sodium citrate, in 100 ml water, pH 7) at room temperature for 5 min each, and then 2X SSC at 37°C for 5 min. Sections were fixed 10 min with 10% buffered formalin to minimize diffusion of amplicons from the cell during hybridization, washed with DPBS, and dehydrated 5 min with absolute ethanol; 20 μl of hybridization mix was added to each slide, covered with a circular glass coverslip (18–22 mm diameter) and placed into the in situ thermocycler. Denaturation was at 95°C for 5 min for cells, 10 min for tissue, and hybridization at 37°C overnight. To detect hybridized labeled probe, sections were gently rinsed with DPBS, endogenous peroxidase quenched with 3% H_2_O_2_ for 10 min, slides washed with DPBS, then soaked with 1% bovine serum albumin (BSA) in 2x SSC for 15 min at 37°C. The reaction was developed by adding 1:100 anti-dig-POD antibody to sections, and visualizing with the chromagen diaminobenzidine (DAB) (Sigma). Outcome measurement was a semi-quantitative light microscopic judgment of brown color density of the cellular reactions (1+–4+).

Specimens were scored positive for the presence of BLV DNA amplicons only if all the following conditions were met: 1) positive and negative cell line controls had the appropriate reactions, confirmed and pictured in our previous publication [6]; 2) cellular reactions were ≥ 2+ rating; 3) BLV amplicons were found in mammary epithelium; and 4) the background control for non-specific tissue reactions (an adjacent section from the same tissue reacted with ISH reagents minus the labeled probe) was negative for the corresponding area. Specimens were scored independently by two us (GCB and HMS), blinded to breast cancer status.

For sequencing, DNA was extracted using the QiAmp DNA mini kit (Qiagin). Primer sequences, below, were from the LTR region of the BLV genome as shown below, with bp numbering according to GenBank accession #EF600696:

Outer primers:

Forward (bp 23–38): TAGGAGCCGCCACCGC

Reverse (bp 352–336): GCGGTGGTCTCAGCCGA

Inner primers:

Forward (bp 41–59): CGTAAACCAGACAGAGACG

Reverse (bp 331–312): CACCCTCCAAACCGTGCTTG

Using thin walled GeneMate PCR tubes (BioExpress, Kaysville, UT, USA), sample DNA (50ng) was added to 50 μL of PCR mix (2.0 mmol/L MgCl_2_, 0.2 mmol/L dNTPs, 0.025 U/μL Taq polymerase [all from Promega, Madison, WI, USA], and 0.2 μmol/L outer primers for each BLV gene. For the second (nested) round of amplification, 2 μL of the first-round PCR product was added to a new tube of the same reaction mix with inner primers shown above. Cycling conditions for outer primers were: 1 cycle at 95°C for 2 min; 35 cycles of 95°C for 30s, 57°C for 30s, 72°C for 24s, then 1 cycle of 72°C for 5 min. Cycling conditions for inner primers were: 1 cycle at 95°C for 2 min; 35 cycles of 95°C for 30s, 58°C for 30s, 72°C for 22s, then 1 cycle of 72°C for 5 min. Reaction mix and conditions, including MgCl2 concentrations, were previously optimized using the BLV-positive FLK cell line and BLV-negative Tb_1_Lu cell line [6]. Sensitivity of the nested L-PCR was previously determined by using the housekeeping gene GAPDH, which occurs in humans as 1 copy/cell [6].

BLV amplicons obtained by nested PCR were separated by electrophoreses in 1.5% agarose gel at 100 V, excised from the gel, and cleaned using Zymoclean Gel DNA Recovery Kit (Zymo Research, Irvine, CA, USA). The requested sample (100 ng/1000 bp length of DNA in 12 μL water and 1 μL of the 5 μmol/L sequencing primer stock solution of the inner primer pair for each genome region) was submitted to the University of California, Berkeley, DNA Sequencing Facility for direct sequencing. DNA for sequencing was obtained from 2–5 amplifications and sequenced at least once in forward (5′) and reverse (3′) directions. Sequences were checked against corresponding electropherograms. Variations from the reference sequence (GenBank accession no. EF600696) were considered valid only if they matched in both forward and reverse directions. Targeted sequences were relatively short because formalin causes DNA breaks, making it difficult to obtain sequences >300 bp [14].

Statistical analysis of age differences between cases versus controls and virus-positive versus virus-negative subjects used two-sided Student t-tests. The STATA “cc” function for case-control data was used to compute unadjusted odds ratio and 95% confidence interval (using Cornfield’s approximation) for association between BLV status and breast cancer [15]. For subjects with paired specimens, the later specimen was used for the case-control data. Unconditional multivariate logistic regression was used to determine age-adjusted odds ratio for association between breast cancer and BLV. No adjustment was needed for catchment area, as all subjects were from the greater Sydney area, or for race/ethnicity because this information was not provided with the specimens. For analyses of paired specimens from the same subject, contingency tables were prepared using Microsoft EXCEL to examine concordance of BLV status in the specimen taken at Time 1 versus the specimen at Time 2. Time interval between the first and second surgery of each donor was compared between subjects who remained cancer-free versus subjects who developed cancer, using a two-sided Student t-test and Fisher's exact test for the contingency table.

## Results

**Fig 1:**
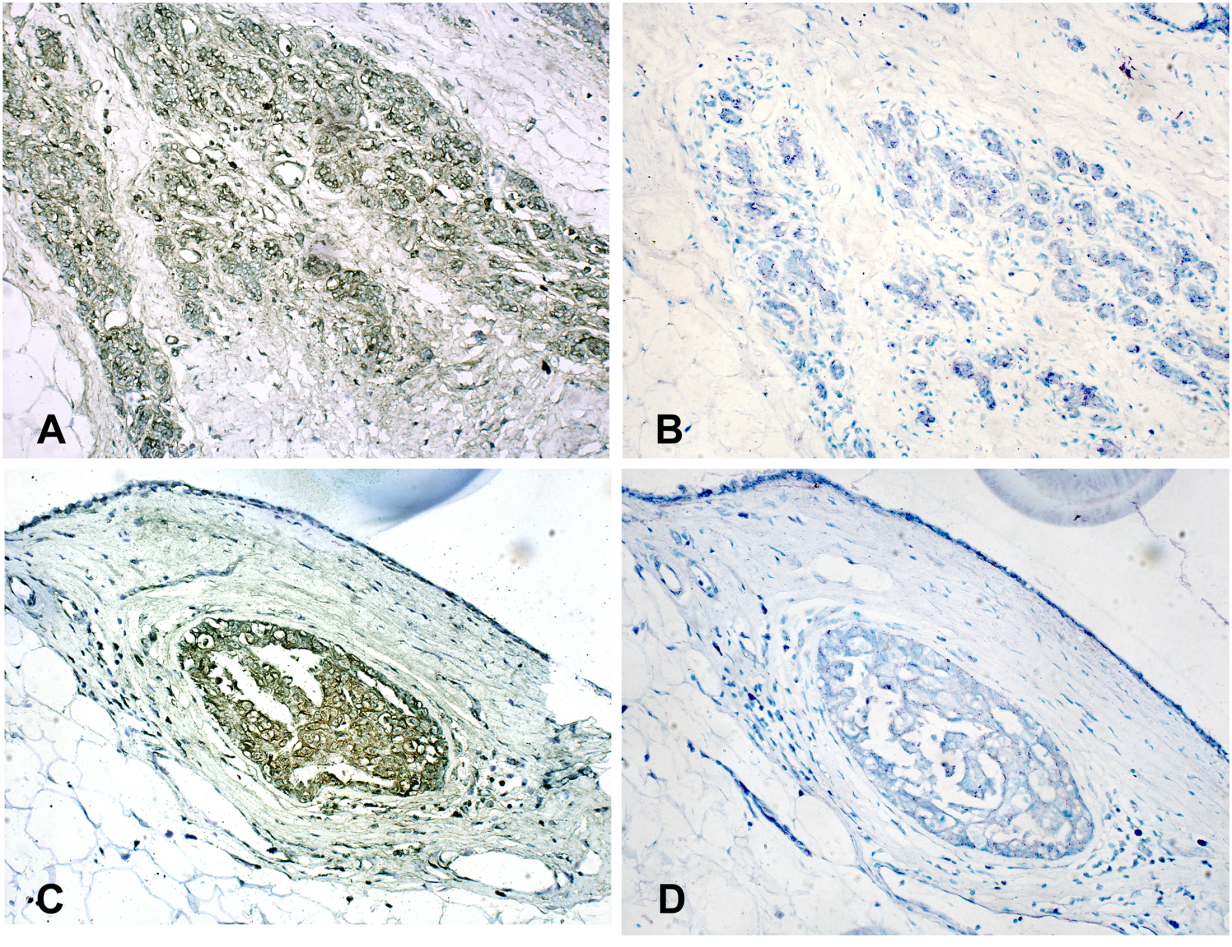
BLV-specific DNA in human breast tissue amplified and detected by PCR-in situ hybridization. A) Breast tissue specimen diagnosed as invasive pleomorphic lobular carcinoma. Three streaks of BLV-positive (brown staining) mammary epithelial cells are seen invading through connective tissue (fibroblasts and adipocytes) that is largely BLV-negative. Positive reactions in mammary epithelial cells are in cytoplasm (diffuse light brown reaction) of most mammary epithelial cells, and nuclei of some cells (diffuse brown reaction as well as dark dots) and as compared to B) Adjacent tissue section without the BLV-specific ISH probe in the hybridization mix. This serves as a background control against which to compare A, and also a control for reactions due to artifacts inherent in some tissues (excess peroxidase and/or melanin). No brown cells are apparent in B. C) Breast tissue specimen diagnosed as benign ductal hyperplasia (normal). Note oval nest of BLV-positive mammary epithelial cells tightly surrounded by fibroblasts that are BLV-negative. Positive reactions in the mammary epithelial cells are intense in the cytoplasm and some cells have evidence of positive nuclear reactions (dark brown dots). D) Background control for C has no apparent brown cells. Light counterstain for all specimens illustrated is Difquick blue and the magnification is x40.

Among this population of Australian women, BLV was present in mammary epithelial cells of 59/96 (61.5%) subjects. Fig 1A illustrates positive reactions in malignant mammary epithelial cells in a specimen diagnosed as invasive pleiomorphic lobular carcinoma. Fig 1B illustrates the background control, an adjacent section of each tissue analyzed in hybridization solution minus the labeled probe, to check for the artifact of brown color inherent in some tissues, e.g. melanin pigment, or high levels of endogenous peroxidase, especially in phagocytic cells. Fig 1C illustrates positive reactions in nonmalignant (benign) mammary epithelium in a specimen diagnosed as ductal hyperplasia, and Fig 1D its background control. There was no conspicuous morphologic difference between individual BLV-infected versus uninfected mammary epithelial cells within breast tissues of any diagnosis. Regardless of the pathology of the mammary epithelial cells in a specimen, BLV-positive cells were almost exclusively found as part of a population of like cells in an area such as duct or a lobule, rather than as single cells scattered among BLV-negative cells.

**Fig 2:**
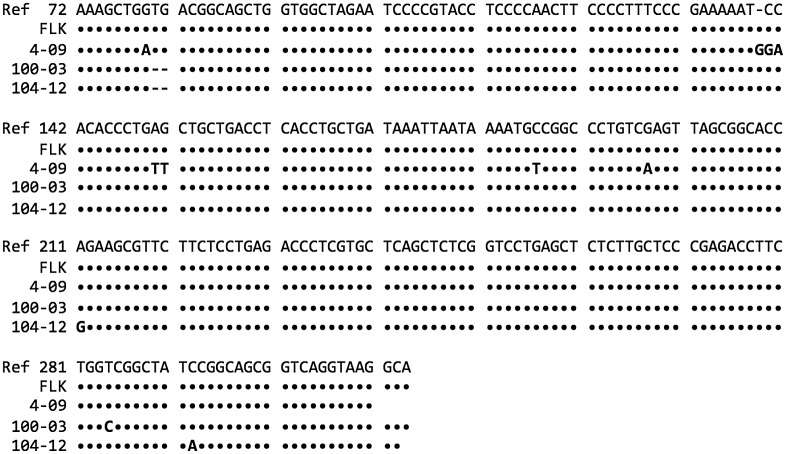
BLV-specific DNA sequences of the viral long terminal repeat (LTR) promoter region. Sequences from breast tissues of 3 subjects (4–09, 100–3, 104–12) are compared to the reference sequence (Ref) (GenBank accession no. EF600696) and the positive control cell line FLK. Base pair position in the reference sequence is indicated by the number following “Ref”. Dots indicate a match with the reference sequence. Variations from the reference sequence are indicated by the uppercase letters for the substituting base. A hypen (-) indicates a deletion at that position. A space indicates the sequence was not obtained beyond that point.

Additional evidence that the virus detected was BLV was obtained for 14 specimens that had sufficient material for DNA extraction and sequencing. The sequences of the positive control cell line and 10 of the subjects’ sequences matched the reference sequence exactly for the span of the LTR examined. The sequences of 4 subjects contained at least one base substitution for this segment of the LTR examined. These are shown in Fig 2.

A case-control study design was used to examine the association between exposure to BLV and the development of breast cancer. Detection of BLV DNA in mammary epithelial cells, the cell type from which most breast cancers arise, served as a biomarker of BLV infection (exposure) and a diagnosis of breast cancer or premalignant breast disease served as the measure of disease outcome. Statistical analysis indicated an initial unadjusted odds ratio (OR) of 5.68, but since the age-range of the subjects at the time of surgery was 16–83 years and the mean and median ages of cases and controls were significantly different (Table 1), age-adjustment was necessary. The age-adjusted OR for risk of developing breast cancer if exposed to BLV was 4.72 with a confidence interval (CI) of 1.71–13.05 (Table 2, complete data sheet in S1 Table).

**Table 1:** Relationship of age of subjects to case/control status and BLV presence*.

	All subjects (n = 96)	Cases (n = 50)	Controls (n = 46)	p	Subjects BLV+ (n = 59)	Subjects BLV- (n = 37)	p
**Age range**	16–83	34–83	16–79		19–77	16–83	
**Mean (SD)**	49.59 (14.64)	55.08 (13.73)	42.43 (14.53)	≤0.001	52.54 (13.13)	44.89 (15.84)	≤0.012
** Median**	48	53	42.5		52	46	

*BLV = bovine leukemia virus; SD = standard deviation; p = probability

**Table 2:** Association of bovine leukemia virus presence in tissue with diagnosis of breast cancer (unadjusted and age-adjusted odds ratios and confidence intervals)*.

Virus Status	Diagnosis of breast cancer			Unadjusted odds ratio (95% CI)	p	Age-adjusted odds ratio (95% CI)	p
	All subjects	Cases	Controls				
**BLV+**	59 (61.5%)	40 (80.0%)	19 (41.3%)	5.68 (2.11–15.75)	≤0.0001	4.72 (1.71–13.05)	≤0.003
**BLV-**	37 (38.5%)	10 (20.0%)	27 (58.7%)				

*BLV = bovine leukemia virus; CI = confidence interval; OR = odds ratio; p = probability

To test whether temporal exposure to BLV preceded development of breast cancer, paired specimens from individual subjects were analyzed. For 48 subjects, an archived normal breast tissue was available from breast surgery performed 3–10 years previously (S2 Table). The time interval between specimens did not differ significantly between women who developed cancer versus women with no breast cancer history (Table 3). The probability of developing breast cancer was significantly higher in women whose breast epithelial cells contained BLV in both the first and the second specimen (Table 4, Virus Status, column 1, marginal frequency = 60.4%) versus in women whose specimens showed no BLV in both first and second specimen, or BLV in only one of the two paired specimens (Table 4, Virus Status, columns 2,3,4; total marginal frequency = 39.6%).

**Table 3:** Time interval between paired specimens in women who developed versus did not develop breast cancer (BCa) after the first specimen, which was benign*.

	Breast Cancer Status		
Time interval (years)	Developed BCa (n = 28)	Didn’t develop BCa (n = 20)	p
**Mean (SD)**	6.86 (2.27)	5.75 (2.22)	0.10
**Median**	6.5	5	

*BCa = breast cancer; SD = standard deviation; n = number of subjects, p = probability

**Table 4:** Probability of developing breast cancer in women whose breast epithelial cells contained BLV in both the first and second specimen versus women whose breast epithelial cells were BLV+ in only one of the paired specimens*.

		Viral Status				Marginal Frequency
		BLV+ →BLV+	BLV+ →BLV-	BLV- →BLV+	BLV- →BLV-	
Breast Pathology	**B →M**	20	2	5	1	28/48 (58.3%)
	**PM → M**	3	0	0	0	3/48 (6.3%)
	**B →B**	6	7	2	2	17/48 (35.4%)
Marginal Frequency		29/48 (60.4%)	9/48 (18.8%)	7/48(14.6%)	3/48 (6.3%)	48

Pr (B **→** M | BLV+ → BLV+/ BLV+ → BLV = 20/29 = 0.69 Fisher’s exact test p = 0.0484

*B = benign; BLV = bovine leukemia virus; M = malignant; PM = premalignant; + = BLV present in tissue; − = no BLV present in tissue; **→,** time progression pointing toward second specimen; Pr = probability;

## Discussion

For the past ≈30 years, BLV has not been regarded as a threat to humans, e.g. “BLV is not transmissible to humans, and no human disease has ever been attributed to BLV.” [16]. However, in a previous study, we were able to demonstrate retrotranscribed BLV DNA by in situ PCR in breast tissues from 97/219 (44%) of US women [6]. These results were validated by in situ PCR performed on the 6 specimens submitted to an independent laboratory at Ohio State University, blinded to the pathology classification of the tissue and our results [6]. BLV genome segments were also detected in breast tissue of Columbian [17] women. In one collaborative lung tumor study by the Moffitt Cancer Center, Tampa, Florida and the Lawrence Livermore National Laboratory, Livermore, California, BLV DNA was detected in 8/10(80%) squamous cell carcinomas of the lung using next generation sequencing and a reference sequence data base designed to detect all sequenced viral and bacterial families [18,19]. These studies reopen the issue of BLV’s transmissibility to humans.

Results of this case-control study in Australian women from the greater Sydney area were consistent with results for US women [7], detecting BLV significantly more frequently in breast tissues of breast cancer patients versus women with no history of breast cancer. However, Australian women had a higher overall frequency of BLV in breast tissue compared to US women, both in breast cancer patients (77.6% versus 59%) and women with no history of breast cancer (41.3% versus 29%). Age-adjusted odds ratio and confidence interval for the Australian women was also much higher (4.72 [1.71–13.05]) than for the US women (3.1 [1.66–5.69]). One possible explanation for this difference is that the sample size of the Australian women was much smaller (n = 96) than for the US women (n = 214) and may not have been representative. Another explanation is that the Australian population may have had a higher proportion of women of European ancestry than the US population. Although we received no information about the race/ethnicity of the Australian subjects, the US population studied consisted of 73% women of European ancestry and 26% women of African ancestry, the latter of which had a far lower frequency (29%) of BLV in their breast tissue than did the women of European ancestry (50%). There is as yet no explanation for the lower incidence of BLV-related breast cancer among the African-American subjects, but one possibility is that their consumption of standard dairy products is lower than that of European-Americans (14% versus 22% of total protein consumed) [20].

One limitation of this study is the sample size, constrained by the scarcity of paired breast tissue specimens from individual donors. This resulted in a small sample size for each of the individual categories presented in Table 4, which might have reduced the power of the statistical analysis. The minimum 3-year interval, however, was a strength of the study in assuring that the condition that prompted the initial surgery (usually reduction mammoplasty or a biopsy that turned out to be benign) was not immediately related to the malignancy that necessitated the second surgery. Other strengths of the study include the sensitivity of the PCR-ISH method and its utility in enabling identification of the specific cell type harboring BLV DNA, in this case mammary epithelial cells, the cell type from which most breast cancers arise. In addition, PCR-ISH is resistant to molecular contamination, as the in situ PCR mix has a different composition from that used for standard liquid PCR; it amplifies only DNA fixed into tissues and will not amplify extraneous DNA molecules that might accidentally enter the PCR mix. The use of primers from the highly conserved *tax* region [21] for the PCR-ISH enhanced the chances of detecting BLV, since it has been shown in cattle that the *gag*, *pol*, and *env* regions, coding for capsid protein, polymerase, and envelope protein, respectively, frequently become deleted as the malignancy progresses, but *tax* deletion occurs less frequently [22].

Primers used for DNA sequencing were from the BLV LTR promoter region, also rarely deleted during tumor progression, but having more sequence variation than the *tax* region. Sequencing indicated a few base substitution polymorphisms, which differed from each other and from the positive control cell line, suggesting that cross-contamination was unlikely. Standard liquid phase PCR (L-PCR), which requires initial tissue digestion to extract DNA, is prone to molecular contamination. We reduced this probability by best practices described previously in detail [6]. Also, the use of a BLV-infected sheep cell line as the source of our positive control BLV DNA, enabled us to check our human DNA samples for contamination with DNA from the positive control cell line by using species specific primers that amplify the ovine cytochrome C oxidase I gene [12].

A concern occasionally raised in discussions of virally caused human cancers is that presence of any virus in the breast may not relate to causation of breast cancer, but instead could represent an opportunistic virus invasion of tissue already transformed to malignancy, with virus presence enhanced by the already accelerated division of the malignant cells. In this study, we were able to demonstrate, using paired breast tissues from the same donor removed at least 3 years apart, that in 23/31 (74.2%) subjects diagnosed with breast cancer, BLV infection of benign or premalignant breast tissue was present years before the breast cancer diagnosis (Fisher exact test result of p≤ .05). This argues against the idea of viral invasion of already malignant cells. While this does not prove BLV causation of breast cancer, it is consistent with a potential role of BLV in initiating the malignant process rather than infecting already malignant tissue.

Table 4 summarizes the details of the 12 different categories resulting from comparing disease progression from benign or premalignant to malignant with the BLV status in first versus second of the paired specimens. Although there are several comparative analyses that could be pursued based on the data in Table 4, we chose to compare samples with BLV in both first and second specimen from each donor, with all other categories combined for several reasons: 1) A longer, continuous presence of BLV in the breast (both specimens BLV-positive) would be more likely to result in malignant transformation of the cells, as the process of progression to malignancy has been estimated to take decades [23]. 2) Women whose specimens went from BLV negative to positive may have acquired the virus too close to the second surgery to allow time for BLV to play a major role in breast cancer initiation/causation. 3) Women whose BLV status went from positive to negative may have cleared the virus completely and therefore could represent a population less vulnerable to disease caused by BLV. This situation is illustrated by human papilloma virus (HPV) infection of the uterine cervix; in a study of 4825 women, >90% of those whose cervix tested positive for oncogenic HPV types cleared the virus completely over several months to a year and never developed cervical cancer [24].

It is not known how humans acquire infection with BLV. It could be from other humans already infected with BLV or from consumption of unpasteurized dairy or undercooked beef products from BLV infected cattle. Only about 5% of BLV infected cattle develop lymphosarcoma, and in most countries their products cannot be marketed. Although the remaining 95% of infected cattle may be marketed, they have lower milk production and incur increased costs of diagnostic testing. These economic issues, and the potential loss of international markets due to the success of the BLV eradication program in the European Union, provided an incentive for Australian farmers to voluntarily progress toward a statewide and eventually nationwide program for the eradication of BLV in dairy herds [25]. The program was begun in 1997, and in 2012, BLV was declared officially eradicated (infection rate ≤0.5%) in dairy herds of Australia [25,26]. The specimens used in this study were from surgeries performed 1996–2013, and therefore, the subjects had potential exposure to a bovine source of BLV that predates eradication from Australian herds. Will the BLV eradication program result in a future decline of human breast cancer? The latency period (time interval between exposure to an oncogenic virus and appearance of cancer) is an estimated 20–50 years based on studies of human T-cell leukemia virus, human papilloma virus (cancer of the uterine cervix), and hepatitis B virus (hepatocellular carcinoma) [23]. If the same latency periods holds true for BLV in humans, it could be a few decades before any decline in breast cancer due to BLV eradication in Australian cattle would be apparent, but it is worth waiting for.

## Conclusions

Bovine leukemia virus is significantly associated with breast cancer in a population of Australian women, and was present in some breast tissues 3–10 years before the cancer was diagnosed.

## Supporting information

S1 File Online primary data on unpaired specimens. (Buehring et al. AustBLPLOSoneUnpairedData).
(XLSX)

S2 File Online primary data on paired specimens. (Buehring et al. AustBLV,PLOSonePairedData).
(XLSX)
